# Fourier transform mid infrared spectroscopy applications for monitoring the structural plasticity of plant cell walls

**DOI:** 10.3389/fpls.2014.00303

**Published:** 2014-06-30

**Authors:** Asier Largo-Gosens, Mabel Hernández-Altamirano, Laura García-Calvo, Ana Alonso-Simón, Jesús Álvarez, José L. Acebes

**Affiliations:** Área de Fisiología Vegetal, Departamento de Ingeniería y Ciencias Agrarias, Facultad de Ciencias Biológicas y Ambientales, Universidad de León, LeónSpain

**Keywords:** cell wall, stress, mutants, development, FT-MIR spectroscopy

## Abstract

Fourier transform mid-infrared (FT-MIR) spectroscopy has been extensively used as a potent, fast and non-destructive procedure for analyzing cell wall architectures, with the capacity to provide abundant information about their polymers, functional groups, and *in muro* entanglement. In conjunction with multivariate analyses, this method has proved to be a valuable tool for tracking alterations in cell walls. The present review examines recent progress in the use of FT-MIR spectroscopy to monitor cell wall changes occurring *in muro* as a result of various factors, such as growth and development processes, genetic modifications, exposition or habituation to cellulose biosynthesis inhibitors and responses to other abiotic or biotic stresses, as well as its biotechnological applications.

## INTRODUCTION

The plant cell wall is a very complex structure, mainly formed by carbohydrates and proteins, which surrounds the plant’s protoplasts. This structure not only determines a cell’s size and shape, but also provides protection against stresses and biological damage (Seifert and Blaukopf, 2010).

The importance of plant cell wall is twofold: on the one hand, it has many applications for human society, providing raw material which can be processed to make textiles, paper, wood, livestock feed, dietary fiber, fuel, etc. (Harris et al., 2010). On the other hand, its structure is incredibly resistant to both physical and chemical stresses while at the same time being extremely plastic, endowing cells with the capacity to adapt to different situations by modifying its composition and structure (Cheung and Wu, 2011). Therefore, knowledge of the mechanisms by which such adaptations occur could facilitate the design of cell walls with compositions adapted to different specific uses.

However, its complex structure and composition, as well as its resistance, render the plant cell wall a difficult target to study. There are many different molecules in its structure which may be linked with different kind of bonds, from weaker hydrogen bonds to stronger ester o even ether bonds. Moreover, all these molecules are arranged in different network which are not totally independent from each other (Fry, 2011). Thus, the main scaffold of plant cell wall is formed by cellulose microfibrils, linked to hemicelluloses by hydrogen bonds. This cellulose-hemicellulose network is embedded in a gel of matrix pectins, including simple and more complex homogalacturonans, rhamnogalacturonans, arabinans, galactans, and arabinogalactans. Several structural proteins and a set of enzymes also form part of the plant cell wall (Carpita and Gibeaut, 1993).

**FIGURE 1:**
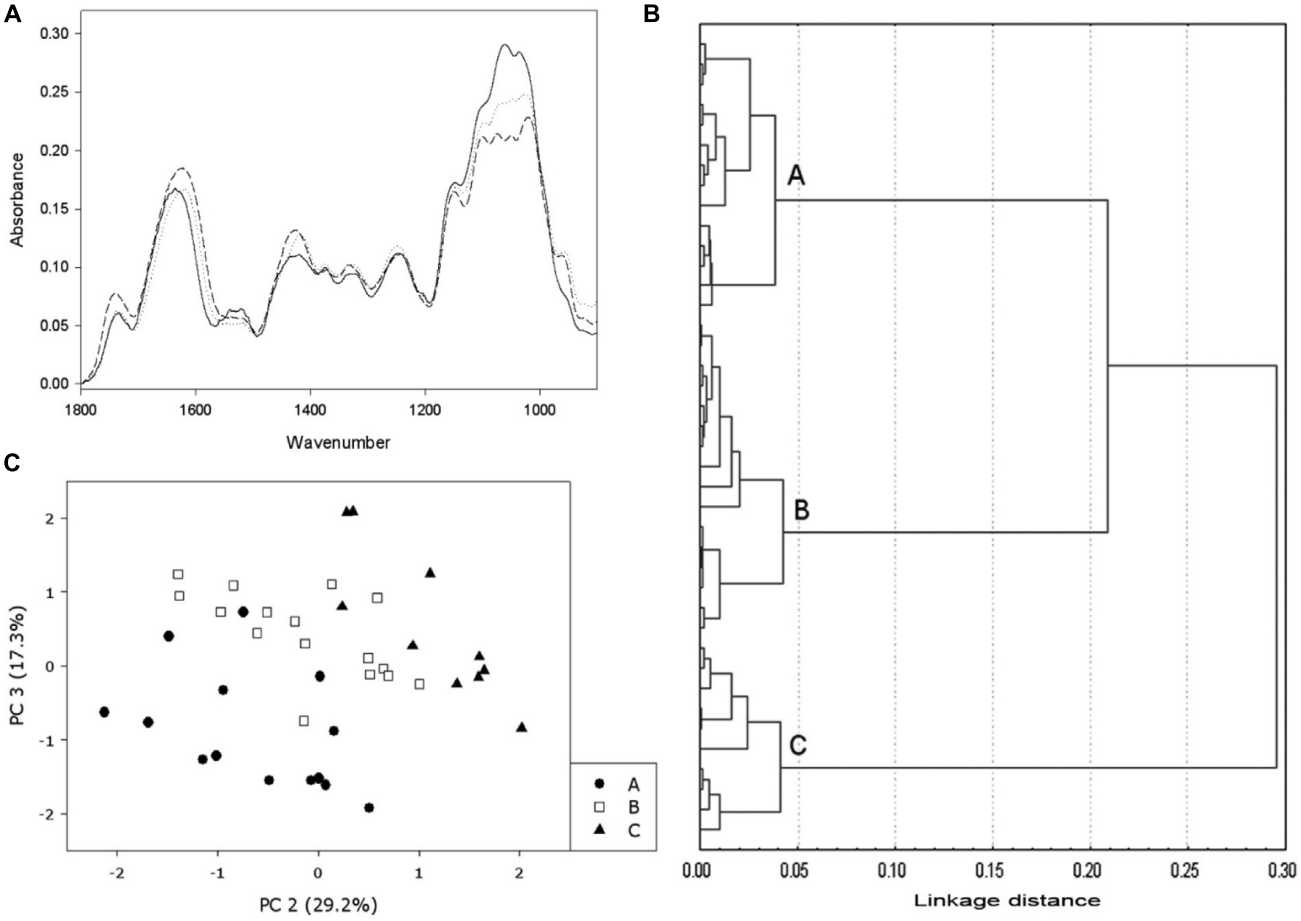
**Example of FT-MIR use in the monitorization of DCB-habituated cells. (A)** FT-MIR spectra from different types of cells: non-habituated cells (solid line), low level of habituation (dotted line) and high level of habituation (dashed line). **(B)** Cluster analysis performed on several spectra from non-habituated cells and DCB-habituated cells, growing on concentrations ranging from 0.5 to 12 μM; the analysis made three different groups: non-habituated (group A), low level of habituation (group B) and high level of habituation to DCB (group C). **(C)** Principal Component Analysis (PCA) performed on the same bulk of spectra. The same groups of cluster analysis can be observed. (Data from Alonso-Simón et al., 2004).

As a result of its structural complexity, research into the plant cell wall is extremely challenging, and even more so given how time-consuming it is to isolate and subsequently extract and fractionate its different components. These procedures require large amounts of sample and involve the use of contaminant solvents and harsh conditions, which may alter the native structure of plant cell wall components during isolation. Thus, fourier transform mid-infrared (FT-MIR) spectroscopy (also known as FTIR spectroscopy) has been extensively used to analyze plant cell walls (Dokken et al., 2005; Alonso-Simón et al., 2011). In contrast with other analytical methods, FT-MIR analysis is a fast procedure that provides information of polysaccharides *in muro*, without the need to extract or solubilize- and therefore alter- any cell wall component (**Figure 1**). The chemical specificity of mid-infrared region allows identification of certain peaks related to cell wall components (Smith-Moritz et al., 2011; **Table 1**). Moreover, carbohydrates show high absorbance in the 1,200–950 cm^-1^ region, known as the fingerprint region, where the position and intensity of the bands is specific for each polysaccharide (Oliveira et al., 2009). In addition, this technique only requires a small amount of sample, and may even be combined to optical microscopy (FT-MIR microspectroscopy) to analyze small areas of the plant cell wall. In this case, a microscope accessory is attached to the FTIR device in such a way that the infrared beam from the spectrometer is diverted to pass through a sample placed in the microscope stage. Thus, the sample can be mapped, moving it under computer control such that different areas of the sample are measured in turn, generating an array of spectra. These spectra can be correlated with visual images of the sample, so that optically observed features can be associated with functional groups (McCann et al., 2001).

**Table 1:** A summary of assignment of wave numbers obtained by FT-MIR spectroscopy to main cell wall components.

Assigned cell wall component	Wavenumber (cm^-1^)	Reference
Protein	1550, 1650	Séné et al. (1994), Wang et al. (2009b)
Pectins	952, 1014, 1097, 1104, 1146, 1243	Coimbra et al. (1999), Kacuráková et al. (2000)
De-esterified pectins (carboxylic acid).	1420, 1600–1630	Mouille et al. (2003)
Esterified uronic acid	1740	McCann et al. (1992)
Pectin’s acetylester	1017, 1047, 1100	Gou et al. (2008)
Lignin	1510, 1530–1540, 1595	Rodrigues et al. (1998)
Syringyl units of lignin	1330	Faix (1992)
Guaiacyl units of lignin	1270	Faix (1992)
Phenolic compounds	1430	Séné et al. (1994)
Phenolic ring	1515, 1630	Séné et al. (1994), Carpita et al. (2001)
Phenolic ester	1720	Séné et al. (1994), Carpita et al. (2001)
Cellulose	900, 990, 1040, 1060, 1109, 1160, 1320, 1367	Carpita et al. (2001), Brown et al. (2005), Rubio-Díaz et al. (2012)
Xyloglucan	1041, 1078, 1120, 1317, 1371	Coimbra et al. (1999), Kacuráková et al. (2000)
Arabinogalactan	1043, 1078, 1139	Barbosa et al. (2013)
Glycerolipids and wax hydrocarbons	1470, 2850, 2920	Kanter et al. (2013)
Wax- or suberin-like aliphatic compounds	1318, 1372, 1734–1745	Zeier and Schreiber (1999), Wang et al. (2009a)

Besides FT-MIR, which encompasses the wavelength region over the range of 400–4000 cm^-1^ (25000–2500 nm), FT-NIR, which includes the 4000–12000 cm^-1^ wavelength region (700–2500 nm), has also been applied in order to identify plant cell wall variations associated with specific monosaccharide dissimilarities and thus identify cell wall mutants from a potential mutant population (Smith-Moritz et al., 2011).

Despite recent significant improvements, the application of FT-MIR analysis may also present certain limitations due to the huge amount of data included in a single spectrum, and also because of overlapping bands corresponding to different plant cell wall components. The combined use of FT-MIR with statistical tools, mainly multivariate analysis, has improved interpretation of the obtained results, especially when analyzing a large number of spectra (**Figure 1**). Thus, the combination of features such as rapidity, low amount of sample required and the huge amount of data obtained from this analysis, renders FT-MIR in conjunction with multivariate analysis a valuable tool for tracking or monitoring alterations in plant cell walls in response to different situations. In this review, we examine recent use of FT-MIR spectroscopy to monitor plant cell wall modifications occurred not only during processes such as growth and development, but also as a consequence of mutation or overexpression of cell wall-related genes and in response to different kinds of biotic and abiotic stress, paying special attention to exposure or habituation to cellulose biosynthesis inhibitors (CBIs).

## THE USE OF FT-MIR SPECTROSCOPY TO STUDY CHANGES IN CELL WALL COMPOSITION AND ARCHITECTURE RELATED TO GROWTH AND DEVELOPMENT

Over the last two decades, a considerable amount of research has been conducted on the involvement of the cell wall in plant growth and development. FT-MIR has proved to be a useful tool for monitoring and/or corroborating cell wall changes related to diverse stages of plant development.

Fourier transform mid-infrared spectroscopy has been used successfully to characterize growth-related modifications in the composition and structure of a wide range of cell types and cultures. Using this approach, growth throughout the culture cycle of *Arabidopsis* suspension-cultured cells has been elucidated, indicating that the mechanical properties of the cell wall correlate with its composition during cell growth. The main modifications revealed by FT-MIR are a loss of lignin (revealed by a decrease in the band between 1530 and 1540 cm^-1^; **Table 1**) and a predominance of polysaccharides (denoted by increases in the bands at 1040, 1060, 1160, and 1245 cm^-1^) as cell growth progresses (Radotić et al., 2012).

In terms of specific stages of plant cell growth and development, FT-MIR spectroscopy has contributed to characterize changes in cell walls during seed development and germination. More specifically, cell wall differences between hard and soft wheat (*Triticum aestivum*) endosperm textures, as well as cell wall modifications during wheat grain development, have been studied by means of FT-MIR microspectroscopy and subsequent statistical and imaging analysis (Barron et al., 2005). These techniques have demonstrated that the main difference between the two endosperm textures is the presence of higher amounts of a water-extractable arabinoxylan in the peripheral endosperm of soft grains. Furthermore, this technique has revealed marked differences in the ratio of β-1,3-1,4 glucans / arabinoxylans, and in the arabinoxylan structure, depending both on cell position within the endosperm grain and the stage of development (Saulnier et al., 2009).

Similarly, the use of FT-MIR microspectroscopy and statistical analysis to study the involvement of cell wall components in *Arabidopsis* embryo germination has confirmed that inactivation of arabinogalactan proteins using the Yariv reagent induces changes in cell wall composition (an enrichment in cellulose - revealed by peaks at 900 and 1320 cm^-1^ - and an impoverishment in pectins - denoted by a decrease in the peaks at 1014, 1094, 1152, 1238, and 1741 cm^-1^ - leading to cessation of embryo germination and abnormal cotyledon embryos formation (Zhong et al., 2011).

Regarding seedling growth, FT-MIR spectroscopy has been used in order to study the role of the cell wall and its components throughout this process. Since plant coleoptiles have a simple and homogeneous structure, they have frequently been selected to perform this kind of study. Maize (*Zea mays*) coleoptile growth has been investigated with FT-MIR microspectroscopy using a chemical imaging tool and neural networks to classify infrared spectra (Carpita et al., 2001; McCann et al., 2001, 2007). This approach revealed dynamic cell wall changes among tissues of this growing organ, and has determined the importance, from among other cell wall components, of β-1,3-1,4 glucan, and glucomannans during cell elongation (Carpita et al., 2001).

Cell wall modifications during root growth have also been analyzed by means of FT-MIR spectroscopy. The roots of *Cicer arietinum*, *Clivia miniata,* and *Iris germanica* have been used to describe three developmental stages in endodermal cell walls during root growth, related with the deposition of suberin, lignin, cell wall proteins, and carbohydrates in these cells (Zeier and Schreiber, 1999).

In a further study aimed at determining the involvement of cell wall components in relation to salt resistance in developing casparian strips, three cultivars of rice (*Oryza sativa*) with different salt resistance were selected and FT-MIR spectroscopy was used to monitor the accumulation of suberin, lignin, proteins, and polysaccharides during the development of the casparian strips (Cai et al., 2011). The difference FT-MIR spectra revealed that the content of suberin as well as other cell wall component was higher in the resistant rice cultivar than in the two susceptible cultivars (Cai et al., 2011).

Concerning the study of cell wall changes related to the stem development, FT-MIR spectroscopy has been applied to *Brachypodium distachyon* during three developmental stages: elongation, inflorescence emergence, and senescence, revealing that as the stem develops, an accumulation of lignin, crystalline cellulose, and xylan occurs in the cell walls (Matos et al., 2013).

Recently, a combination of FT-MIR microspectroscopy and a focal plane array (FPA) detector, which increments spatial resolution and the data handling by orthogonal projections to latent structures discriminant analysis (OPLS-DA), has enabled the extraction of spectra from single cell types, the characterization of different chemotypes by using the full spectra information rather than only discrete characteristic bands, and the acquisition of chemical landscapes by multivariate analysis (Gorzsás et al., 2011). This technique has been applied to characterize the chemotypes of the different secondary xylem cell types (vessels, fibers, and rays) across the annual wood ring of aspen (*Populus tremula*) and to monitor changes in these cell walls. In fiber cells, lignin was observed to predominate in earlywood and hemicelluloses/cellulose in latewood, which could be explained by the development of an S2 layer of secondary cell wall during the growing season. Similarly, xylem ray cells were found to contain more aromatic compounds (lignin and monolignols) in earlywood and more pectins and/or hemicelluloses in latewood. These findings could be due to changes in the pectin-rich protective layer typical of ray cells. Vessel elements were more uniform throughout the annual ring, probably due to its rapid development (Gorzsás et al., 2011). In another study, FT-MIR microspectroscopy has been useful to monitor the postmortem lignification of tracheary elements of *Zinnia elegans*, revealing the presence of lignin-characteristic bands, as 1510 and 1595 cm^-1^, in the FT-MIR spectra of developing tracheary elements (Pesquet et al., 2013). The exposure of these cells to piperonylic acid, an inhibitor of lignin monomer biosynthesis, induced a reduction in the absorbance on these bands. Moreover, a supply of coniferyl alcohol, or both coniferyl and synapyl alcohols, to the piperonylic acid-treated tracheary elements induced FT-MIR spectra similar to those of non-treated cells (Pesquet et al., 2013).

Pollen tube growth has frequently been studied by means of FT-MIR spectroscopy. Thus, treatment with different toxins that stop pollen tube growth and subsequent analysis of cell wall infrared spectra has revealed that the changes in esterified and acidic pectic distributions and their relative contents are associated with the cessation of pollen tube growth in *Picea wilsonii* (Kong et al., 2006; Sheng et al., 2006; Chen et al., 2008), *Picea meyeri* (Chen et al., 2007), and *Pinus bungeana* (Wang et al., 2009c). The formation of multiple pollen tubes from a single pollen grain has been studied in *Luffa cylindrica*, where FT-MIR microspectroscopy has confirmed that lower amounts of pectins and abnormal cell wall components are deposited in the multiple pollen tubes without nuclei walls (Jiang et al., 2009). These abnormalities were related to lignin, pectin, cellulose, callose and overall cell wall carbohydrate content. Likewise, a study of *Arabidopsis* root hair growth cessation induced by treatment with CdCl_2_ has revealed that cadmium provokes a disruption in vesicle trafficking that affects cell wall deposition and tip growth. The main modifications measured by FT-MIR and immunolabeling of the tip cell wall included a reduction in esterified pectins and an increment in de-esterified pectins, other polysaccharides and proteins (Fan et al., 2011).

Lastly, changes in cell wall composition during fruit development and ripening have been analyzed by FT-MIR spectroscopy to determine the influence of cell wall on fruit development. This is the case of the study of a guaiacyl-syringyl-lignin, which is important during pear (*Pyrus bretschneideri* cv. Dangshan Su) fruit ripening; the use of FT-MIR spectroscopy has made it possible to determine that this lignin has more guaiacyl than syringyl groups in its structure (Cai et al., 2010). FT-MIR spectroscopy has also been used to study the contribution of pectin composition in the ripening of strawberry (*Fragaria x ananassa*) fruits (Posé et al., 2012), and to monitor changes in cell wall composition and cellulose content through the development of cotton (*Gossypium hirsutum*) fibers (Abidi et al., 2014). The main cell wall changes during cotton fiber development consist of a reduction in proteins and pectins, the de-esterification of these pectins, and a huge increment in the cellulose content due to the synthesis of a secondary cell wall (Abidi et al., 2014). Furthermore, FT-MIR spectroscopy has contributed to an analysis of the presence of phenolic compounds (mainly phenolic esters) bound to the cell wall of mature cotton fibers (Fan et al., 2009). FTIR and synchrotron infrared imaging have been used to monitor the acetyl esterification of cell walls of black cotton-wood (*Populus trichocarpa*), showing that *p*-coumarate accumulates in young leaves and declines in mature leaves, while ferulate and acetate are predominantly found in stems. Over the course of stem development, the amount of ferulate increases, whereas the initial amount of *p*-coumarate diminishes (Gou et al., 2008).

Other studies on changes throughout growth and development processes have focused on genetic control of the expression of different kinds of genes, or on the effect of different types of stress on normal plant development, and these will be discussed in the following sections.

## MUTATION AND OVEREXPRESSION OF CELL WALL-RELATED GENES

Fourier transform mid infrared spectroscopy has been used to analyze modifications in cell wall structure and composition due to the mutation or overexpression of genes that presumably have a function in plant cell wall biosynthesis or in its modification. Studies on control of the expression of cell wall-related genes have yielded information about cell wall biosynthesis and turnover of the different cell wall components, thus providing further insights into the function of these genes.

### CELLULOSE BIOSYNTHESIS-RELATED GENES

Cellulose is the major scaffolding polysaccharide in the primary and secondary cell walls of plants, and it is synthesized by the cellulose synthase complexes located at the plasma membrane. These complexes are constituted by six subunits forming a hexagonal rosette. Each subunit is composed of six cellulose synthase (CESA) proteins which synthesize the 36 β-(1,4)-glucan chains that form the cellulose microfibril. However, this model has now been questioned, and 12-36 glucan chains for the microfibril are currently being considered (see McFarlane et al., 2014 for a review). *CesA* genes can be divided in two groups, one of which is related to cellulose biosynthesis of the primary cell wall while the other is related to cellulose biosynthesis of the secondary cell wall (Hamann et al., 2004; McFarlane et al., 2014).

Fourier transform mid infrared spectroscopyhas been applied to discriminate *Arabidopsis* cellulose mutants from a set of characterized and uncharacterized cell wall mutants. For this end, infrared spectra from the cell wall of mutants, and wild type plants treated with the CBI isoxaben were obtained, and a set of statistical tools were used, with the separation and classification of these mutants being visualized by means of a dendrogram (Robin et al., 2003). It was observed that alleles of the same loci were clustered, as were wild type plants treated with low concentrations of isoxaben, whereas the other clustered mutations were those which affected cellulose biosynthesis-related genes as well as the plants treated with high concentrations of isoxaben, which both showed a reduction in cellulose content. The same authors performed a similar study which confirmed the high capacity of these techniques to discriminate mutants with cellulose defects from other cell wall mutants, irrespective of whether they had been characterized or not (Mouille et al., 2003).

Besides this application of FT-MIR, the technique has been used to ascertain the role of different *CesA* genes in cellulose biosynthesis. Thus, to determine the role of CesA6 in cellulose biosynthesis in *Arabidopsis*, non-polarized deuteration-FT-MIR spectroscopy was applied to the *procuste* mutation (*prc-1*), which affects CesA6 expression. This mutant showed a reduction of hypocotyl elongation when it was grown in dark conditions (Fagard et al., 2000; MacKinnon et al., 2006). FT-MIR spectroscopy of *prc-1* cell walls revealed a small reduction in cellulose content, which was accompanied by a relative increase in pectin and altered pectin esterification. Using polarized FT-MIR with FE-SEM-emission-scanning electron microscopy to analyze the orientation of the cellulose microfibrils, it was observed that *prc-1* cellulose microfibrils in elongated cells were similar to wild type cell walls, although less uniform, whereas in short cells of the mutant, the orientation was random, ranging between 0 and 180^∘^ to the cell axes (MacKinnon et al., 2006).

In addition, in order to ascertain the role of *AtCesA2* or *AtCesA5* genes and their relation with AtCesA6, FT-MIR microspectroscopy was applied to cell walls from dark-grown hypocotyls of mutants *cesa2* and *cesa5*, and double mutants *cesa2 cesa5*, *cesa2 cesa6* and *cesa5 cesa6* (Desprez et al., 2007). Spectra of cell walls from *cesa2*, *cesa5* and even *cesa2 cesa5* clustered with those of wild type controls, whereas those of double mutants *cesa2 cesa6* and *cesa5 cesa6* were grouped with *prc 1-1* and other cellulose deficient mutants. These results, combined with others, suggest that CESA2, CESA5, and CESA6 proteins are partially redundant in the cellulose complex (Desprez et al., 2007).

The* thanatos* mutant (*than*) is affected in the secondary structure of the catalytic cytosolic domain of AtCESA3 (Daras et al., 2009). This mutation provokes several phenotypic changes, such as a reduction in plant growth, especially when the mutation is homozygous. FT-MIR analysis of *than* mutant cell walls showed a reduction in cellulose that was corroborated by a strong decline in [^14^C]glucose incorporation into cellulose. Moreover, the cell wall FT-MIR spectra revealed a reduction in saturated ester groups and an increment in proteins and phenolic compounds, indicating ectopic lignin deposition in the mutant (Daras et al., 2009).

Regarding the characterization of cellulose biosynthesis in the secondary cell wall, FT-MIR spectroscopy has also been applied to a set of 16 genes putatively related to secondary cell wall formation (Brown et al., 2005). These were selected by using profiling techniques, and some examples are *irx1* (irregular xylem), *irx3*, and *irx5* mutants, which are caused by defects in the CesA gene family (CesA8, CesA7, and CesA4, respectively), and their proteins form part of the complex involved in cellulose synthesis in secondary cell wall [for a revision see McFarlane et al. (2014)]. FT-MIR and subsequent PCA revealed that these mutants exhibit an important reduction in cellulose content (Brown et al., 2005). Previously, cell walls of *irx3* had been studied using solid-state NMR spectroscopy and FT-MIR microscopy, showing that sclerenchyma tissues of hypocotyls of this mutant contained reduced amounts of crystalline cellulose that did not seem to be replaced by amorphous glucans (Ha et al., 2002).

FT-MIR has also been useful in the characterization of three *exigua* mutants, which are impaired in secondary cellulose biosynthesis: they showed different cell wall changes in the vascular tissue, but all presented a reduced cellulose content concomitant with an increase in lignin (Rubio-Díaz et al., 2012).

In another study, FT-MIR was applied to the ammonium oxalate-extracted mucilage of seeds of the triple *Arabidopsis* mutant *cesa2*/*cesa5*/*cesa9*, revealing differences not only in xyloglucan and pectin composition but also in cellulose, thus indicating not only that cellulose is present in mucilage, but also that cellulose mutants have mucilage with altered composition (Mendu et al., 2011).

In addition to *Arabidopsis*, other species, such as potato (*Solanum tuberosum*) have been used to study *CesA* homolog genes (Oomen et al., 2004). In this case, up- and downregulation of four *CesA* genes was studied with FT-MIR to analyze changes in cellulose as well as overall polysaccharide content in tuber sections, showing different reductions in the cellulose levels of transgenic lines wirh respect to wild type samples.

Besides to *CesA*, other genes have been implicated in cellulose biosynthesis. A point mutation in the KORRIGAN (KOR) gene that encodes a β,1-4 endoglucanase, impairs a reduction in cellulose deposition in secondary cell wall, causing the *Arabidopsis irregular xylem 2* (*irx2*) mutant phenotype (Szyjanowicz et al., 2004). The FT-MIR spectra of deuterated primary cell walls of *irx-2-1* showed that there were apparently no changes either in the crystalline structure of cellulose or in the non-cellulosic polysaccharide composition, compared to wild type cell walls. These results complement other findings on KOR expression and protein immunolocalization, and suggest that KOR might be involved in the processing of growing microfibrils during later stages of secondary cell wall formation or in the release of cellulose synthase complexes (Szyjanowicz et al., 2004).

To conclude this section, another gene whose characterization has been associated with cellulose biosynthesis mutants and to which FT-MIR has been applied, is a plasma-membrane bound receptor-like kinase, THESEUS (THE-1). FT-MIR microspectroscopy and PCA applied to *prc1-1* and the double mutant *the-1/prc1-1* grouped both mutants together, and in the same cluster as other cellulose mutants, indicating that they are cellulose deficient. This finding concurs with other related results, suggesting that the THE1 protein may operate as a cell wall integrity sensor, mediating the response of growing plant cells to perturbations in cellulose synthesis (Hématy et al., 2007).

### HEMICELLULOSE AND PECTIN BIOSYNTHESIS GENES

FT-MIR has also been successfully used to study the biosynthesis or modifications of other cell wall polysaccharides, namely hemicelluloses and pectins.

Xylans are the most abundant hemicelluloses in secondary cell walls, and FT-MIR spectroscopy has proved to be an effective means of characterizing and grouping secondary cell wall mutants. The application of FT-MIR spectroscopy and multivariate analyses to a set of irregular xylem mutants, has enabled characterization of *irx7*, *irx8* and *irx9*, which showed important reductions in xylose content in the inflorescence stem, whereas their cellulose content did not seem to be affected, indicating that the alteration in cell wall composition of these mutants is consistent with a lack of β-1,4-linked xylosyl residues associated with xylan (Brown et al., 2005). Subsequently, FT-MIR was applied to a set of xylan-affected and other secondary cell-wall mutants: *irx14*, *parvus-3*, *irx1*, *irx3*, *irx5*, *irx7*, *irx8* and *irx9* (Brown et al., 2007). A principal components analysis enabled discrimination between cellulose-affected mutants and those affected in xylans: *parvus-3*, *irx7*, *irx8* and *irx9* formed a group, whereas *irx14* formed a single cluster. These data, together with polysaccharide fractionation, cell wall sugar and PACE analyses, show that *irx7*, *irx8* and *irx9* are xylan-deficient mutants (Brown et al., 2007).

FT-MIR was applied in another study to analyze the *irx8* mutant. The results showed that it had potential alterations in non-cellulosic polymers, helping to demonstrate that its dwarf phenotype could not be attributed to a cellulose deficiency. Moreover, sugar analysis and cellulose quantification revealed that it had glucuronoxylan and homogalacturonan deficiencies (Persson et al., 2007). In addition, an extensive analysis using FT-MIR microspectroscopy combined with OPLS-DA demonstrated that the *fra8* (*irx7*) gene in *Arabidopsis* causes a decrease in the proportion of xylan and lignin, and that it is involved in xylan biosynthesis in lignified as well as in non-lignified fibers of the secondary xylem of hypocotyl (Gorzsás et al., 2011).

Further studies have described new mutants affected in xylan composition, and have led to the conclusion that *irx 7*–*9* as well as *parvus* mutants are affected in different glycosyltransferases involved in xylan synthesis (for a review see Pauly et al., 2013 and Jensen et al., 2013).

FT-MIR spectroscopy has also been used to ascertain some of the genes involved in xyloglucan biosynthesis, another of the main hemicellulosic polysaccharides. FT-MIR analysis of the *xxt5* mutant showed that it displays comparable cell wall changes to *xxt1 xxt2* mutant plants (Zabotina et al., 2008), XXT1 and XXT2 having previously being implicated in xyloglucan biosynthesis. Nowadays, the three genes are known to codify xyloxyltransferases involved in xyloglucan biosynthesis (Pauly et al., 2013).

Besides biosynthesis, modification of xyloglucan has also been elucidated using FT-MIR spectroscopy. Xyloglucan endotransglycosylase/hydrolase (XTH) enzymes have been implicated in modification of the xyloglucan structure, and as a consequence, in cell wall reorganization. In tomato plants overexpressing XTH1, FT-MIR cell wall spectra from the apical hypocotyls of these transgenic plants showed that the linkages between pectic polysaccharides, and between xyloglucan and cellulose differ with respect to the wild type, and that this structure could be related to altered cell wall extensibility in their hypocotyls (Miedes et al., 2011).

Regarding pectin biosynthesis and modification, FT-MIR analyses of transgenic plants overexpressing AtRHM1, a gene putatively implied in rhamnose biosynthesis, have revealed that surplus rhamnose upon overexpression is used in the synthesis of rhamnogalacturonan (Wang et al., 2009a). In another study, overexpression of an inhibitor of pectin methylesterase (PMEI4) delayed growth acceleration in *Arabidopsis* hypocotyls. FT-MIR spectroscopy also showed an increase in ester bonds in these transgenic lines, concomitant with reduced pectin methylesterase activity, thus demonstrating a role of pectin methyl esterification in growth control (Pelletier et al., 2010). Related to this, pectin acetylesterase has been shown to play a role in cell wall properties and cell extensibility, controlling the degree of pectin acetylation. FT-MIR has helped to confirm that transgenic black cotton-wood plants overexpressing pectin acetyl esterase 1 (PtPAE1) have a decreased ratio of ester peaks in relation to polysaccharides in style and filament tissues compared with wild type, which is related to decreased cellular elongation in these organs (Gou et al., 2012).

### LIGNIN BIOSYNTHESIS-RELATED GENES

The genetic control of lignin biosynthesis has usually been elucidated by modifying the expression of phenylpropanoid pathway genes, thus disrupting the normal formation of several phenolic compounds. This pathway terminates with the formation of the three monolignols (*p*-coumaryl alcohol, coniferyl alcohol and sinapyl alcohol) which act as a source to synthesize the lignin leading to the three lignin units: the *p*-hydroxyphenyl (H), guaiacyl (G), and syringyl (S) units.

One of the relevant steps of the phenylpropanoid pathway is carried out by a group of enzymes that are classified as *O*-methyltrasferases (OMTs). This group is composed of two principal enzymes: caffeic acid 5-*O*-methyltrasferase (COMT) and caffeoyl coenzyme A 3-*O*-methyltransferase (CCoAOMT), both of which are responsible for the two methylation steps necessary for biosynthesis of the monolignols previous to their incorporation into lignocellulose. By obtaining an *Arabidopsis* CCoAOMT 1 mutant (*ccomt1*), a gene that is only expressed in lignified tissues, and a double mutant with the other OMTs gene, COMT 1 (*ccomt1 comt1*), it has been demonstrated that these enzymes have a redundant function in the synthesis of lignin, flavonoids, and synapoyl malate (Do et al., 2007). FT-MIR microspectroscopy has been used to corroborate the reduction in lignin and the increment in S units caused by the *ccomt1* mutation (Do et al., 2007). However, in woody poplar (*Populus tremula x Populus alba*) the CCoAOMT gene is essential for normal lignin biosynthesis (Zhong et al., 2000). By using an antisense approach against CCoAOMT, a reduction in the expression of this gene induced a decrease in Klason lignin content. The use of diffuse reflectance infrared Fourier transformed spectroscopy (DRIFTS) has corroborated this reduction in lignin, which was less condensed and cross-linked in the transgenic poplar wood (Zhong et al., 2000). Another study of OMTs involvement in lignin biosynthesis was carried out on tobacco (*Nicotiana tabacum*), using antisense transformation to suppress the caffeic/5-hydroxyl-ferulic acid O-methyltransferase, an enzyme involved in synapyl alcohol formation for lignin (Blaschke et al., 2004). Neither analytical measurements nor FT-MIR spectroscopy data revealed any changes in lignin content as a result of this reduced OMT expression. However, FT-MIR spectroscopy of extracted lignin showed that the stem lignin presented a relative increase in G units compared with S units (whose characteristic IR bands are 1270 cm^-1^ and 1330 cm^-1^, respectively) in antisense-OMT tobacco, and that this change in lignin composition was more pronounced under CO_2_ treatment (Blaschke et al., 2004).

Cinnamoyl-coenzyme A reductase (CCR) is the first enzyme in the monolignol biosynthesis branch of the phenylpropanoid pathway. This enzyme catalyzes the conversion of cinnamoyl coA esters to the corresponding cinnamaldehydes. FT-MIR spectroscopy has been used to determine or corroborate changes in the cell wall and lignin composition of plants with a modified CCR expression. Knock-out of the AtCCR1 gene, which controls the constitutive biosynthesis of lignin, yielded dwarfed *Arabidopsis* plants with delayed senescence (Derikvand et al., 2008). FT-MIR microspectroscopy has also been used to study the changes provoked by mutation in the xylem and stem fibers of these plants, confirming the significant reduction in lignin content measured by several analytical techniques. FT-MIR spectra revealed that the fiber region presented a significant reduction in lignin and enrichment in hemicelluloses. There was no detection of lignin reduction in the xylem region, but it showed the same enrichment in hemicelluloses (Derikvand et al., 2008). In the case of woody poplar, downregulation of CCR by antisense transformation induced a reduction in lignin content and the appearance of an orange-brown coloration in the outer xylem (Leplé et al., 2007). This reduction in lignin content was corroborated by both FT-MIR spectroscopy and analytical studies. FT-MIR spectroscopy supported the notion that this mutation increases the breakdown and remodeling of non-cellulosic cell wall polymers and decreased biosynthesis, mainly of the hemicelluloses. Furthermore, an analysis of the spectra obtained revealed that ferulic acid had been incorporated into the cell wall (Leplé et al., 2007).

The last step of monolignols biosynthesis, prior to polymerization into the lignocellulose molecule, is catalyzed by cinnamyl alcohol dehydrogenase (CAD), converting the cinnamaldehydes into their corresponding alcohols. In *Arabidopsis* floral stems, *CAD-C* and *CAD-D* genes are the primary genes involved in lignin biosynthesis. A FT-MIR spectroscopy study of the double mutant *cad-c cad-d* revealed that the repercussions on xylem and fiber cell wall composition were different. The mutation provoked a reduction in lignin content and a higher amount of G units in their structure, especially in xylem fibers. Furthermore, FT-MIR analysis corroborated an enhancement of cinnamaldehydes incorporated into the cell wall of both cell types (Sibout et al., 2005). Similarly, an antisense transformation of full length cDNA from the CAD gene in the woody plant *Eucalyptus camaldulensis* did not induce any change in lignin content observable by FT-MIR, nor in lignin composition by pirolysis. Moreover, no significant differences in monosaccharide content were detected when studied by FT-MIR using reference standards (Valério et al., 2003).

These studies provide evidence of the existence of direct genetic control of the expression of phenylpropanoid pathway genes. In addition, control of the expression of peroxidases, enzymes necessary for oxidative coupling of the monolignols to synthesize the lignin molecule, has also yielded information about this lignin polymerization process. A study of the *Arabidopsis AtPrx72* knock-out mutant, a gene which is homologous to the *Zinnia elegans ZePrx* gene that has been related to lignin biosynthesis, revealed a decrease in lignin content and in the S moieties of this molecule that has been confirmed by FT-MIR spectroscopy (Herrero et al., 2013). In addition, another strategy is the analysis of a transcription factor that regulates the expression of different genes of this pathway and therefore also regulates lignin biosynthesis. One group of transcription factors is the MYB family. Within this group, *Arabidopsis* MYB103 transcription factor indirectly regulates lignin biosynthesis (Öhman et al., 2013). MYB103 is a member of a transcriptional network that regulates secondary cell wall biosynthesis in the xylem of *Arabidopsis* and it is related to cellulose biosynthesis. Suppression by means of t-DNA insertion of this gene induced a huge reduction in the expression of the ferulate 5-hydroxylase gene (F5H, a member of the phenylpropanoid pathway). Interestingly, lignin content was not affected, and FT-MIR microspectroscopy study of the vessel elements, xylem fibers and inter-fascicular fibers of the inflorescence stem revealed a higher proportion of G units in the lignin of all cell types of the mutant plants (Öhman et al., 2013).

### OTHER MUTANTS WITH MODIFIED CELL WALLS

Lastly, the cell walls of a wide range of mutants or gene-overexpressing plants have been shown to be affected. The exact gene affected is frequently unknown, but a clear quantitative or qualitative modification in their cell wall composition has been found. In some of these plants FT-MIR has been used to characterize their cell walls. Some examples include the rolling leaf mutant (*rlm*) of rice, where FT-MIR spectra from foliar cell walls showed lower protein and polysaccharide contents (Bai et al., 2008); the root-specific α-expansin gene of rice, OsExpa8, where FT-MIR of cell walls of plants overexpressing this gene displayed increased ratios of polysaccharide/lignin content, therefore supporting a role for expansins in cell elongation and plant growth (Ma et al., 2013); or the *shaven3* (*shv3*) mutant of *Arabidopsis*, whose encoded protein SHV3 has two tandem repeating glycerophosphoryl diester phosphodiesterase-like domains and a glycosylphosphatidylinositol anchor, where FT-MIR of cell walls from the double mutant *shv3* and the paralog *svl1* revealed an alteration in cellulose content and modification in pectins (Hayashi et al., 2008).

## ANALYSIS OF EFFECTS OF BIOTIC AND ABIOTIC STRESS ON THE CELL WALL BY MEANS OF FT-MIR

In nature, plants are exposed to diverse environmental stress factors, and the responses of plants to these stresses involve changes at physiological, biochemical and molecular levels. Abiotic stress factors, such as heat, cold, drought, salinity, presence of heavy metals, or poor nutrition, have a worldwide impact on agriculture, since they present a serious threat to crop production. Besides these abiotic factors, biotic stress factors such as fungi, bacteria, virus, nematodes, and herbivores also compromise plant survival (Wang et al., 2003; Atkinson and Urwin, 2012). As the cell wall is normally the first line of defense at cellular level against all of these stresses, technologies such as FT-MIR spectroscopy have frequently been used in order to monitor changes in this structure due to stress factors.

Among the diverse sources of stress, drought is the most important factor limiting plant growth, reproductive development and, ultimately, survival (Arbona et al., 2013). Desiccation tolerance in poikilohydric plants is related to changes in the structure and composition of the cell wall (Vicré et al., 2004; Moore et al., 2006). The dried state of desiccation-tolerant tissues limits the type of technique that can be applied to study conformation and stability of biomolecules. One of the few suitable techniques for dried tissue analysis is FT-MIR spectroscopy, because it can be used irrespective of the hydration state of the tissue (Wolkers and Hoekstra, 2003). Species such as *Boea hygrometrica* – a desiccation-tolerant angiosperm (or resurrection plant) – have been selected as models to investigate changes in gene expression and cell wall adaptation during extreme dehydration, and morphological changes such as smooth cell wall folding have been observed in cell wall architecture during the process of drying and re-watering. With regard to the chemical composition of the leaf cell wall, the FT-MIR spectra indicated that protein levels rose upon desiccation and remained at those levels after re-watering (Wang et al., 2009b), whereas an increase of both esterified and de-esterified pectins was observed in rehydrated leaves. The absorbance levels at wavenumbers assigned to phenolics showed no differences in lignin or monolignols between hydrated and dehydrated leaves. Lastly, the intensity of peaks corresponding to octadecyl octadecanoate indicated that wax or suberin-like aliphatic compounds increased in rehydrated leaves (Wang et al., 2009a).

Regarding nutrient deficiency studies, callus-cultured cells of vine (*Vitis vinifera*) were subject to nitrogen, phosphorous and sulfur impairment, and changes in their cell walls were analyzed with FT-MIR spectroscopy (Fernandes et al., 2013). This study showed differences in cellulose content when comparing control and sulfur deficient (with nitrogen) and phosphorous deficient calluses. Nitrogen deficient calluses exhibited a lower amount of cellulose and proteins, and a higher amount of pectins.

Heat stress also causes alterations in cell wall components (Hasanuzzaman et al., 2013). When leaves of coffee plants were grown at 37^∘^C, important changes in their cell walls were identified: the pectic content was decreased by almost 50% in the cell wall of heat stressed leaves (Barbosa et al., 2013). Based upon the FT-MIR spectra of water-soluble polysaccharides, temperatures of 37^∘^C induce a higher content of arabinose and galactose and a reduced content of mannose, glucose, uronic acid, rhamnose, and fucose. In the hemicellulosic fractions, the main components were arabinoxylans and xyloglucans; and the xylose content was decreased under heat stress (Barbosa et al., 2013).

Fruit is usually stored at low temperatures in order to delay ripening; however, tropical fruits are susceptible to chilling injury and this is the cause of extensive post-harvest losses. Chilling injury damage involves alteration in the properties of the cell walls. In one study, mango (*Mangifera indica* L. cv. “Red 6”) fruits were subjected to low-temperature stress and then analyzed by histochemical and scanning electron microscopy together with FT-MIR. The results showed that chilling injury symptoms (sunken lesions, alterations in the outer pericarp, and changes in cuticular waxes and cell walls) were limited in fruit treated with methyl salicylate as compared to the 5^∘^C control (Han et al., 2006), demonstrating the positive effects of this compound as regards conferring tolerance to low-temperature stress (Fung et al., 2004). A FT-MIR spectrometric analysis indicated high proportions of linear long-chain aliphatic, phenolic rings and pectic polysaccharides in cell wall extracts but lower amounts of cellulose, in mango fruit stored at 5^∘^C with methyl salicylate (Han et al., 2006).

Air pollution in general and ozone in particular affects plants, altering their growth and pollen production intensity (Booker et al., 2009). Furthermore, ozone seems to affect both the morphology of pollen (size, shape, surface structure) and the abundance of allergenic protein (Kanter et al., 2013). FT-MIR has shown that pollen samples exposed to elevated ozone levels exhibit a reduction in phenolic compounds. The reduction in absorbance of the FT-MIR peaks corresponding to acetyl ester of pectin in fumigated pollen was consistent with expressed sequence tags data. At the same time, an increased pectic content was observed, hence indicating that de-esterification could be an effect of ozone treatment (Kanter et al., 2013).

Plant responses to biotic stresses involve physiological and biochemical changes, including activation of the expression of defense-related genes, whose function is to protect the plant against these kinds of stresses (Hamann, 2012). FT-MIR analysis of *pmr5* (powdery mildew resistant) *Arabidopsis* plants, which possess a mutation of unknown function that nevertheless confers protection against powdery mildew infection, showed an increment in pectin in their cell walls, and that these pectins had a lower degree of methyl esterification or O-acetylation (Vogel et al., 2004), basically coinciding with the FT-MIR spectra of the mutant *pmr6*, affected in a glycosylphosphatidylinositol-anchored pectate lyase-like gene (Vogel et al., 2002). Other mutants affected in different subunits of the heterotrimeric G-proteins, such as *agb1* and the double mutant *agg1 agg2*, exhibited similar FT-MIR spectra to each other, but different from those of wild type, suggesting that G-protein subunits play a role in the control of cell wall composition and in the immune response of *Arabidopsis* plants (Delgado-Cerezo et al., 2012).

By means of FT-MIR spectroscopy it has been possible to monitor the changes in wood elm (*Ulmus minor*) biochemistry as a consequence of fungi infection by *Ophiostoma novo-*ulmi (Martín et al., 2005). Lower levels of polysaccharides and higher levels of phenolic and aliphatic compounds in xylem tissues of inoculated elms were found, suggesting that cell wall degradation occurred as a result of fungal enzyme activity. The high levels of aliphatic compounds could be related to the presence of tyloses and to the suberization of parenchyma cell walls. A prominent negative peak at 983 cm^-1^ was also found, suggesting a higher starch content in resistant elm trees; thus, a reduction in starch should be associated with tree susceptibility to infection (Martín et al., 2005). FT-MIR combined with PCA has therefore proved to be a valid means of monitoring plant-pathogen interaction and identifying resistant genotypes, a function that could be applied to other species.

FT-MIR spectroscopy has also been used to detect cell wall changes provoked by infection with *Verticillium longisporum*: the main difference found between non-infected and infected cells occurred in the degree of pectin esterification (Floerl et al., 2012). When the effects of a combination of biotic and abiotic stresses (infection with *Phaeomoniella chlamydospora* and cell exposure to NaCl) were monitored in stems, leaves and roots of grapevine using FT-MIR spectroscopy, major changes were observed in cells exposed to both stresses (Oliveira et al., 2009). The spectra of leaves subjected to both stresses were characterized by higher amounts of pectic polysaccharides, xylose-rich polysaccharides and cellulose.

## FT-MIR MONITORING OF CELL WALL CHANGES ASSOCIATED WITH CBIs

CBIs are a heterogeneous group of compounds with the capacity to interfere with cellulose biosynthesis (for a review, see Acebes et al., 2010). FT-MIR spectroscopy has been applied in conjunction with multivariate analyses to monitor cell wall changes associated to CBIs, related either to (i) short-term exposure of plant cell cultures or complete plants to CBIs, or (ii) habituation/dehabituation of plant cell cultures to these kinds of compound.

Some studies have applied FT-MIR spectroscopy in order to characterize the effect of short-term exposure to CBIs on cell wall structure and composition, such as the study carried out by Peng et al. (2013) on *Arabidopsis* seedlings treated with 2,6-dichlorobenzonitrile (DCB), a well-known CBI. The authors used FT-MIR spectroscopy to confirm the reduction in cellulose and pectin content in the cell wall previously observed with S4B (a specific fluorescence dye for cellulose) staining, or JIM5 (a monoclonal antibody that recognizes relatively unesterified pectic epitopes) immunolabeling. Moreover, FT-MIR spectra also showed a huge increase in protein in the cell wall of treated seedlings (Peng et al., 2013).

Another set of experiments related to a short-term exposure to CBIs was conducted in a recent study of the role of cellulose biosynthesis in the polarized growth of *Pinus bungeana* pollen tubes (Hao et al., 2013). Inhibition of cellulose biosynthesis by means of a DCB treatment induced a dose-dependent change in the growth rate and morphology of pollen tubes by altering the chemical composition of the tube wall. These cell wall modifications, initially characterized with fluorochromes and monoclonal antibodies, consisted of the accumulation of callose and pectins in the tips of the pollen tube and a decreased content of cellulose. A comparison between the FT-MIR spectra of control pollen tubes and the spectra of pollen tubes treated with DCB revealed changes in absorbance intensity and location of specific peaks, further confirmed by chemical and immunolabeling analyses. The difference spectra generated by digital subtraction of the control spectra from those of DCB-treated pollen tubes revealed that the saturated ester peak and the amide stretches increased, while cellulose content distinctly decreased with increasing DCB concentrations.

FT-MIR has also been used to analyze the cell wall composition of the *Arabidopsis* mutant *aegeus* (*cesa1^aegeus^*), which shows resistance to the CBI quinoxyphen (Harris et al., 2012). In this case, the *aegeus* mutant displayed semi-dominant inheritance similar to that observed for the isoxaben resistance mutant *cesa3^ixr1-2^*. It has been suggested that quinoxyphen and isoxaben share a common mechanism of action on cellulose biosynthesis, and further analysis of quinoxyphen-exposed seedlings has revealed a hyper-accumulation of callose and ectopic lignin production, modifications that have also been observed with other CBIs (Harris et al., 2012).

FT-MIR microspectroscopy and PCA were applied to screen *Arabidopsis* mutants deficient and non-deficient in cellulose, and to compare them with the wild type treated with a variety of CBIs, such as DCB, isoxaben, thaxtomin A and flupoxam (Mouille et al., 2003). Two main branches were distinguished in the resulting dendrogram: one branch grouped cellulose deficient mutants and wild type treated with a high concentration of CBIs, while the other branch contained untreated wild type, or wild type treated with a low concentration of CBI, together with the mutants not affected in cellulose content. The alignment of the spectra corresponding to the wild type treated with thaxtomin A was one of the first pieces of evidence supporting the idea that thaxtomin A inhibits the synthesis of cellulose, which has also been confirmed by chemical analysis (Scheible et al., 2003).

The second group of experiments included habituating cell cultures to grow in the presence of CBIs. Initially, tomato cultured cells (with a type I cell wall) were habituated to DCB and were characterized by biochemical or immunolabeling techniques and later compared with data from FT-MIR spectroscopy (Shedletzky et al., 1990; Wells et al., 1994). These analyses of DCB-habituated cells showed a unique cell wall with drastically reduced levels of cellulose, where the major load-bearing network of the cell wall was formed by calcium-linked pectins. Immunogold labeling with the JIM5 antibody showed that pectins from the habituated cell walls were mainly unesterified. Further characterization of the cells walls by FT-MIR spectroscopy yielded additional evidence that supported the previous biochemical and immunogold-labeling studies (Wells et al., 1994).

Bean (*Phaseolus vulgaris*) calluses (also with cells surrounded by type I cell wall) were habituated to grow in a relatively high DCB concentration (12 μM) and then characterized (Encina et al., 2001). Cell wall isolation and fractionation and the subsequent chemical analyses showed that the xyloglucan–cellulose network of non-habituated cell walls was partly replaced in DCB-habituated cell walls by a pectin-rich network mainly formed of cross-linked polyuronides with a large proportion of homogalacturonan. These modifications were comparable to those described for bean calluses habituated to isoxaben (Díaz-Cacho et al., 1999), suggesting a common mechanism of habituation to both CBIs. A comparison of the FT-MIR spectra obtained from the cell walls of DCB-habituated and non-habituated calluses showed that the former had higher peaks associated with ester linkages and an increase in carboxylic acid stretching. Accordingly, the difference spectrum (DCB-habituated cells minus non-habituated cells) showed peaks for ester linkages, free carboxylic groups and uronic acids. Globally, these FT-MIR results confirmed data obtained with chemical and immunological analyses of the entire or fractionated cell walls of DCB-habituated cell lines, showing an increase in both esterified and non-esterified pectins.

Multivariate analysis applied to the FT-MIR spectra data has been a useful technique to monitor further cell wall changes during the habituation of bean calluses to DCB (Alonso-Simón et al., 2004). A dendrogram obtained by cluster analysis of the spectra showed three main branches corresponding to different levels of habituation to DCB: (i) non-habituated calluses and low level habituated calluses (calluses habituated to up to 0.5 μM DCB and calluses with a low number of subcultures in a low concentration of DCB), (ii) medium level habituated calluses (calluses habituated from 0.5 to 4 μM DCB with more than 13 subcultures), and (iii) high level habituated calluses (calluses grown in the highest concentrations of DCB). Principal components revealed that the separation of the above three groups of spectra was associated with peaks related to cellulose and pectins.

In a further study, bean cell suspensions instead of calluses were progressively habituated to DCB, and their cell wall modifications were monitored using a combination of techniques, including FT-MIR spectroscopy. The FT-MIR spectra showed that cell suspensions underwent an increasing enrichment in pectins as the level of habituation to DCB became greater (Encina et al., 2002). When DCB-habituated cells were returned to a medium lacking the herbicide (dehabituation), the observed changes in the FT-MIR spectra were partially reversed, and by the eighth subculture in absence of DCB, spectra resembled those of non-habituated cells (Encina et al., 2002). Long-term dehabituated cells (more than a hundred subcultures in absence of DCB) showed that habituated cells restored cellulose and xyloglucan levels to the cell wall and decreased their pectin content, and that only subtle differences regarding non-habituated cells remained (García-Angulo et al., 2009a). A supplementary study involving FT-MIR spectroscopy proved that some of the cell wall changes induced in bean suspension-cultured cells during habituation were different from those induced in cells during dehabituation, and therefore that habituation and dehabituation follow diverse pathways: PCA indicated that dehabituated cells had more pectins and that these displayed a lower degree of methyl esterification than those of habituated ones (García-Angulo et al., 2009b).

FT-MIR spectroscopy has been also applied in conjunction with PCA to monitor changes in the type II cell wall during habituation of maize cultures to DCB (Mélida et al., 2009). The FT-MIR spectra of walls of DCB-habituated cell lines showed differences in wavenumbers attributed to cellulose, phenolic components, arabinose, and proteins, with respect to non-habituated cells.

More recently, FT-MIR spectroscopy was applied to monitor early changes in cell walls from maize suspension-cultured cells, associated with the first stages of DCB-habituation (de Castro et al., 2014). The difference spectra obtained for each low-habituated cell line compared with the non-habituated cell line showed decreased peaks in wavenumbers attributed to cellulose, together with changes in other peaks associated with phenols and pectins. The changes associated with this incipient habituation tended to revert and this reversion depended on the concentration of the inhibitor and the length of period in contact with it (de Castro et al., 2014).

## ASSESSING BIOTECHNOLOGICAL APPLICATIONS OF CELL WALL MODIFICATIONS

The plant cell wall is the most abundant renewable resource on the planet (Pauly and Keegstra, 2008) and many studies over the last decade have focused on cell wall polymers because they are a source of reduced carbon, which can be used to produce “green fuels.” This material can be obtained from plant wastes that are produced in large amounts by sectors such as the forestry, pulp and paper industries, and can be used as a substrate for ethanol production (Pauly and Keegstra, 2010; Bhatia et al., 2012). Despite the considerable economic interest of the cell walls of plant wastes, only about 2% of this resource is currently used by humans (Pauly and Keegstra, 2008). Cell walls of plant wastes have been analyzed by several techniques, including FT-MIR spectroscopy (Lupoi et al., 2014 and references therein).

The process of transforming lignocellulosic biomass into ethanol requires pretreatment in order to release the mono- or oligosaccharides which can then be fermented into ethanol from larger molecules, such as cellulose or hemicelluloses. Using FT-MIR spectroscopy, glucose, mannose, xylose, and acetic acid has been rapidly quantified in liquors from dilute-acid-pretreated soft-wood and hard-wood slurries in the batch reactor during optimization of pretreatment conditions (Tucker et al., 2000).

In order to transform plant cell walls, it is necessary to use saccharification technology to break down the recalcitrant bonds and release the fermentable sugars. Lignocellulose is the main component that must be processed throughout chemical and enzymatic hydrolysis. To analyze its composition, FT-MIR spectroscopy has been used in combination with PLS (partial least squares) regression to predict sugar production from enzymatic hydrolysis. Six different lignocellulosic raw biomasses pretreated with several levels of NaOH have been analyzed, and FT-MIR spectroscopy has made it possible to describe the solubilization of biomass components (glucose, xylose, lignin) in the fingerprint region of 800–1800 cm^-1^ (Sills and Gossett, 2012).

Regarding ethanol production, a screening strategy has been developed based on stalk geometry and PLS predictive models of FT-MIR spectra collected from soluble sugars and cell wall fractions in *Sorghum bicolor* bagasse. Feedstocks were assayed for total fermentable sugar yields including stalk biomass, soluble sugar concentrations and cell wall saccharification. This method enables prediction of enzymatic cell wall digestibility, and it has been incorporated into a holistic high-throughput screen in a biofuels context (Martin et al., 2013).

Other waste types, such as sugarcane bagasse and coconut fiber, have been used to characterize their main constituents and their behavior in thermal degradation. In both cases, the infrared absorption spectra were related to the presence of lignin, hemicellulose and cellulose, characteristic of natural fibers. Both thermoanalytical and FT-MIR techniques have shown that sugarcane bagasse has a higher thermal stability than coconut fiber (Mothé and de Miranda, 2009).

Similarly, studies have been conducted on the intra-specific variability of lignin and energy contents in extractive-free wood of hybrid poplar progenies (*Populus trichocarpa* × *deltoides*), to determine whether the range is sufficient for the development of quantitative prediction models based on FT-MIR (Zhou et al., 2011). In addition, some timber species such as *Dipterocarpus kerrii*, *Hopea plagata*, *Parashorea malaanoman*, *Shorea almon*, and *Shorea contorta* have been compared, focusing on their durability and potential applications. The observed peaks in wood represent major cell wall components such as cellulose, hemicelluloses and lignin. FT-MIR spectroscopic assays of wood and isolated lignin from *D. kerrii* and *H. plagata* have revealed differences with respect to *P. malaanoman* and *Shorea* sp., species with a short service life. FT-MIR spectra identified lignin composition and ligno-protein content as the principal sources of variation (Rana et al., 2010).

Considering the importance of cell walls in agricultural research, it is surprising that relatively few crop species have benefited from the tools available to rapidly profile their wall polysaccharides (Nguema-Ona et al., 2012). However, FT-MIR has recently been applied to optimize and implement a rapid analysis of the cell wall composition and structure of grapevine leaves. A combination of high-throughput techniques were used, including monosaccharide compositional analysis and FT-MIR spectroscopy, thereby obtaining a rapid profile of their wall polysaccharides (Moore et al., 2014). Similarly, variations in cell wall composition have been monitored by FT-MIR spectroscopy in stems and leaves of a series of *Miscanthus* species and genotypes, at different stages of development, in order to determine their possible application in programs aimed at improving this bioenergy feedstock (da Costa et al., 2014). Variations in cell wall composition reside mainly in the contribution of secondary walls in the stages of peak biomass and after senescence, in comparison to actively growing organs.

FT-MIR analysis of polysaccharide residues from the cell walls of fruits and vegetables such as tomato, potato, pumpkin, carrot and celery root has been used to evaluate differences among cell wall residues and among species, showing that discrimination between the cell wall residues of fruits and vegetables is feasible with the use of FT-MIR spectra in the regions 1800–1200 cm^-1^ and 1200–850 cm^-1^, combined with PCA (Szymanska-Chargot and Zdunek, 2013).

To sum up, FT-MIR spectroscopy has been applied for discrimination of wood from several species, determination of chemical wood composition as well as estimation of ethanol production from renewable agro waste.

## CONCLUDING REMARKS

FT-MIR spectroscopy combined with multivariate analysis is a rapid, non-destructive and easy technique that only requires small amounts of sample and provides abundant information about the “in muro” organization of cell wall polymers and functional groups.

Although FT-MIR has many advantages over traditional analyses of plant cell walls, it is not usually employed in isolation to analyze cell walls, but rather to confirm the results obtained with other chemical or labeling analyses of entire or fractionated cell walls. This is mainly because the information it provides is often incomplete, with limitations derived from the complexity of spectra with overlapping peaks and the vibrational coupling of chemical bonds from different cell wall polymers. Therefore, FT-MIR spectroscopy has been used to monitor cell wall modifications, and to confirm or to complement the data obtained with traditional techniques, such as chemical analyses, fluorescence or immnunolabeling of the entire or fractionated cell walls.

## Conflict of Interest Statement

The authors declare that the research was conducted in the absence of any commercial or financial relationships that could be construed as a potential conflict of interest.
